# Editorial: Mediterranean diet and cancer: Experimental and epidemiological perspectives

**DOI:** 10.3389/fnut.2022.1064503

**Published:** 2022-10-24

**Authors:** Wamidh H. Talib, Mostafa Waly, Eyad Elkord, Lina T. Al Kury

**Affiliations:** ^1^Department of Clinical Pharmacy and Therapeutics, Applied Science Private University, Amman, Jordan; ^2^Department of Food Science and Nutrition, College of Agricultural and Marine Sciences, Sultan Qaboos University, Muscat, Oman; ^3^Natural and Medical Sciences Research Center, University of Nizwa, Nizwa, Oman; ^4^Biomedical Research Center, School of Science, Engineering and Environment, University of Salford, Manchester, United Kingdom; ^5^Department of Health Sciences, College of Natural and Health Sciences, Zayed University, Abu Dhabi, United Arab Emirates

**Keywords:** anticancer diet, alternative therapy, herbal infusion, intermittent fasting, Mediterranean diet

The role of diet in cancer management is gaining more attention and multiple studies suggest nutritional interventions to augment conventional anticancer therapies. Mediterranean diet is one of the healthful dietary patterns. It is composed of many types of foods and drinks rich in antioxidant and biologically active ingredients. This special issue provides new details for researchers, patients, nutritional specialists, and oncologists about the possible role of Mediterranean diet in cancer management.

Consumption of Mediterranean diet is associated with low incidence of breast cancer as shown by Azzeh et al.(a). In this case-control study, researchers concluded that consumption of a diet rich in fruits and vegetables, fish, legumes, black tea, coffee, and low dairy products can significantly reduce the risk of breast cancer [Azzeh et al.(b)]. Spices used in Mediterranean diet were reviewed by Talib et al.. The study showed that black pepper (*Piper nigrum* L.) is the most common spice used in Mediterranean diet. Giger and black cumin were the most active against cancer and apoptosis induction is the most common anticancer mechanism activated by Mediterranean diet spices (Talib et al.). The chemoprevention effect of the Mediterranean diet on colorectal cancer was also investigated by Mahmod et al.. Researchers concluded that components in the Mediterranean diet can reduce the risk of colorectal cancer by reducing inflammation and inhibiting the attachment of pathogenic microbes (Mahmod et al.).

The use of alternative and herbal medicine is increasing among cancer patients. Patients depend on consuming selected herbal infusions or foods containing plant extracts to fight cancer (1). Al-Ataby and Talib showed that daily consumption of lemon and ginger herbal infusion inhibited breast cancer in mice. Phytochemicals in this herbal infusion exhibited high capacity to induce apoptosis, inhibit angiogenesis, and stimulate the immune system (Al-Ataby and Talib). Barley bran grown in Jordan was evaluated by Abuarab and Talib. Results showed anticancer and immunomodulatory effects of barley bran and supported its use as prophylactic agent against cancer (Abuarab and Talib). Aqueous extract of *Elaeagnus* angustifolia flowers inhibited triple-negative breast cancer cells by apoptosis induction as indicted by Fouzat et al.. The anticancer activity of this plant involved activation of P53 and signal transducer and activator of transcription 3 signaling pathways (Fouzat et al.).

Gamal-Eldeen et al. showed that the polysaccharide extract of *Sargassum dentifolium* (an edible brown alga) reduce drug resistance in tongue squamous cell carcinoma by reducing hypoxia.

Gaz-alafi is a local sweet produced mainly in the north of Iraq and west of Iran. Its composition includes secretions from insects and plant products produced from the infected *Quercus brantii* leaves (2). Al Safi et al. showed that aqueous and ethanol extracts of Gaz-alafi are rich in phytochemicals that have anticancer and immunomodulatory effect. Extracts caused regression in tumor growth and stimulation of innate and acquired immunity (Al Safi et al.).

The special issue also discussed the role of intermittent fasting combined with plant extracts to overcome drug resistance. Intermittent fasting is a type of a calorie restriction and involves fasting for 16–48 h. Such fasting stimulates multiple anticancer mechanisms and cause cancer regression (3). Jawarneh and Talib concluded that a combination of Ashwagandha (*Withania somnifera*) root extract and intermittent fasting acts synergistically to overcome cisplatin drug resistance in breast cancer.

Overall, articles included in this issue present a comprehensive scientific contribution to support the use of components in the Mediterranean diet as anticancer nutritional interventions. The special issue also presents the successful use of Mediterranean diet in different combinations to inhibit cancer and reduce drug resistance.

## Author contributions

All authors listed have made a substantial, direct, and intellectual contribution to the work and approved it for publication.

## Conflict of interest

The authors declare that the research was conducted in the absence of any commercial or financial relationships that could be construed as a potential conflict of interest.

## Publisher's note

All claims expressed in this article are solely those of the authors and do not necessarily represent those of their affiliated organizations, or those of the publisher, the editors and the reviewers. Any product that may be evaluated in this article, or claim that may be made by its manufacturer, is not guaranteed or endorsed by the publisher.
